# Supplementary material for: Improving access and efficiency of acute ischemic stroke treatment across four Canadian provinces: a stepped-wedge trial
Source: Front Neurol. 2025 Aug 18;16:1610307. doi: 10.3389/fneur.2025.1610307 (PMC12400858; doi:10.3389/fneur.2025.1610307)
Supplement: Supplementary file 1 [file Supplementary_file_1.docx]

**Supplemental Tables**

Table S1 – Treatment times and outcome measures for all community onset ischemic stroke patients treated with thrombolysis and/or endovascular thrombectomy with sub-group analysis with cases with completed NIHSS, where adjustments were made for cluster, age, and sex. IQR: interquartile range, min: minutes.

|  | **Pre-intervention period** | | **Post-intervention period** | | **Significance** |
| --- | --- | --- | --- | --- | --- |
|  | **n** | **Values** | **n** | **Value** |  |
| Door to CT (min), median (IQR) (unadjusted) | 464 | 17.0 (9.0-36.2) | 580 | 17.0 (10.0-28.0) | 0.80 |
| *Door to Needle (min), median (IQR) (unadjusted)* | *416* | *73.0 (49.0-109.0)* | *566* | *62.5 (40.0-93.0)* | *< 0.0001* |
| Door-in-door-out (min), median (IQR) (unadjusted) | 55 | 148.0 (116.0-189.5) | 91 | 155.5 (127.0-188.5) | 0.62 |
| Door-to-arterial-access (min), median (IQR) (unadjusted) | 106 | 58 (11.2-101.7) | 178 | 70.0 (30.0-100.0) | 0.11 |
| 911-to-needle (min), median (IQR) (unadjusted) | 330 | 129.6 (97.0-164.6) | 413 | 125.0 (93.0-157.0) | 0.14 |
| 911-to-arterial-access (min), median (IQR) (unadjusted) | 69 | 270.0 (147.0-356.0) | 88 | 273.0 (160.0-349.0) | 0.96 |
| Length of Stay^&^ (days) median (IQR) (unadjusted) | 423 | 6.0 (3.0-13.0) | 520 | 6.0 (3.0-14.0) | 0.30 |
| Discharged home, n (%) (unadjusted) | 528 | 272 (51.5%) | 673 | 282 (41.9%) | 0.001 |
| Discharged to rehabilitation, n (%) (unadjusted) | 528 | 64 (12.1%) | 673 | 107 (15.8%) | 0.07 |
| Mortality at discharge, n (%) (unadjusted) | 528 | 84 (15.9%) | 673 | 121 (17.9%) | 0.40 |
| **Subgroup analysis with cases with completed NIHSS** | | | | | |
| Door to CT (min), median (IQR) (unadjusted) | 202 | 17.0 (8.0-39.0) | 217 | 17.0 (10.0-27.0) | 0.33 |
| Door to CT (min), median (IQR) (adjusted) | 202 | 21.6 (6.67-36.4) | 217 | 22.8 (10.15-35.5) | 0.86 |
| Door to Needle (min), median (IQR) (unadjusted) | 161 | 60.0 (43.0-100.0) | 195 | 70.0 (49.0-95.5) | 0.41 |
| Door to Needle (min), median (IQR) (adjusted) | 161 | 74.1 (28.3-120.0) | 195 | 78.9 (41.0-117.0) | 0.006 |
| Door-in-door-out (min), median (IQR) (unadjusted) | 42 | 109.0 (85.0-141.0) | 71 | 130.0 (87.0-178.7) | 0.22 |
| Door-in-door-out (min), median (IQR) (adjusted) | 42 | 119.0 (88.8-150.0) | 71 | 128.0 (84.1-173.0) | 0.20 |
| Door-to-arterial-access (min), median (IQR) (unadjusted) | *91* | *50.0 (11.0-97.5)* | *91* | *30.0 (13.0-95.0)* | *0.01* |
| Door-to-arterial-access (min), median (IQR) (adjusted) | 91 | *59.9 (16.21-103.6)* | *91* | *54.6 (9.8-99.3)* | <0.001 |
| 911-to-needle (min), median (IQR) (unadjusted) | 123 | 113.5 (89.0-158.0) | 160 | 131.3 (104.1-165.0) | 0.02 |
| 911-to-needle (min), median (IQR) (adjusted) | 123 | 117.0 (76.6-156.0) | 160 | 125.0 (87.6-162.0) | 0.33 |
| 911-to-arterial-access (min), median (IQR) (unadjusted) | 61 | 266.0 (147.0-356.0) | 63 | 309.0 (195.0-355.0) | 0.11 |
| 911-to-arterial-access (min), median (IQR) (adjusted) | 61 | 266.0 (244.0-289.0) | 63 | 304.0 (292.0-316.0) | <0.001 |
| Length of Stay^&^ (days) median (IQR) (unadjusted) | 224 | 6.0 (3.0-13.0) | 237 | 5.0 (2.0-16.0) | 0.54 |
| Length of Stay^&^ (days) median (IQR) (adjusted) | 224 | 5.7 (5.6-6.2) | 237 | 6.1 (5.8-6.5) | 0.07 |
| Discharged home, n (%) (unadjusted) | 230 | 111 (48.2%) | 243 | 87 (35.8%) | 0.008 |
| Discharged home, n (%) (adjusted) | 230 | 116 (50.4%) | 243 | 80 (32.9%) | 0.001 |
| Discharged to rehabilitation, n (%) (unadjusted) | 230 | 29 (12.6%) | 243 | 43 (17.6%) | 0.15 |
| Discharged to rehabilitation, n (%) (adjusted) | 230 | 13 (5.6%) | 243 | 15 (6.2%) | 0.84 |
| Mortality at discharge, n (%) (unadjusted) | 230 | 34 (14.7%) | 243 | 47 (19.3%) | 0.23 |
| Mortality at discharge, n(%) (adjusted) | 230 | 26 (11.3%) | 243 | 38 (15.6%) | 0.16 |

Table S-2: Cluster 1 - Treatment times and outcome measures for all community onset ischemic stroke patients treated with thrombolysis and/or endovascular thrombectomy. Adjustments were made for cluster, age, and sex. IQR: interquartile range, min: minutes.

|  | **Pre-intervention period** | | **Post-intervention period** | | **Significance** |
| --- | --- | --- | --- | --- | --- |
|  | **n** | **Values** | **n** | **Value** |  |
| Door to CT (min), median (IQR) (unadjusted) | 125 | 13.0 (8.0-21.0) | 341 | 15 .0 (8.0-22.0) | 0.23 |
| Door to CT (min), median (IQR) (adjusted) | 125 | 13.0 (11.1-14.9) | 341 | 15.0 (13.7-16.3) | 0.32 |
| Door to Needle (min), median (IQR) (unadjusted) | 122 | 57.0 (41.5-83.5) | 346 | 58.0 (37.0-83.0) | 0.79 |
| Door to Needle (min), median (IQR) (adjusted) | 122 | 58.0 (51.7-64.2) | 346 | 58.4 (54.4-62.4) | 0.89 |
| Door-in-door-out (min), median (IQR) (unadjusted) | 19 | 137.0 (114.18-212) | 49 | 153.0 (134.0-187.0) | 0.62 |
| Door-in-door-out (min), median (IQR) (adjusted) | 19 | 163.0 (122-203.0) | 49 | 152 (133.0-172.0) | 0.44 |
| Door-to-arterial-access (min), median (IQR) (unadjusted) | 37 | 86.0 (56.0-123.0) | 106 | 77.5 (63.0-101.8) | 0.42 |
| Door-to-arterial-access (min), median (IQR) (adjusted) | 37 | 85.1 (62.4-107.9) | 106 | 75.7 (68.0-83.5) | 0.21 |
| 911-to-needle (min), median (IQR) (unadjusted) | 102 | 113.9 (89.5-145.3) | 279 | 119.2 (93.8-153.3) | 0.30 |
| 911-to-needle, median (IQR) (adjusted) | 102 | 112.0 (98.1-126.0) | 279 | 118.0 (110.8-125) | 0.42 |
| 911-to-arterial-access (min), median (IQR) (unadjusted) | 15 | 213.0 (146.1-332.1) | 43 | 180.0 (135.9-349.1) | 0.67 |
| 911-to-arterial-access (min), median (IQR) (adjusted) | 15 | 182.0 (80.4-283.0) | 43 | 180.0 (94.0-266.0) | 0.97 |
| Length of Stay (days), median (IQR)^&^ (unadjusted) | 114 | 6 (3-14) | 300 | 6 (3-13) | 0.67 |
| Length of Stay (days), median (IQR)^&^ (adjusted) | 114 | 5.2 (3.7-6.6) | 300 | 6.3 (5.4-7.1) | 0.16 |
| Discharged home, n (%) (unadjusted) | 145 | 65 (44.8%) | 401 | 177 (44.1%) | 0.80 |
| Discharged home, n (%) (adjusted) | 145 | 66 (45.5%) | 401 | 175 (43.6%) | 0.66 |
| Discharged to rehabilitation, n (%) | 145 | 30 (20.6%) | 401 | 77 (19.2%) | 0.79 |
| Discharged to rehabilitation, n (%) (adjusted) | 145 | 31 (21.3%) | 401 | 76 (18.9%) | 0.60 |
| Mortality at discharge, n (%) (unadjusted) | 145 | 27 (18.6%) | 401 | 81( 20.1%) | 0.68 |
| Mortality at discharge, n (%) (adjusted) | 145 | 26 (17.9%) | 401 | 82 (20.4%) | 0.50 |

^&^LOS does **not include** patients that died in hospital

Table S-3: Cluster 2 - Treatment times and outcome measures for all community onset ischemic stroke patients treated with thrombolysis and/or endovascular thrombectomy. Adjustments were made for cluster, age, and sex. IQR: interquartile range, min: minutes.

|  | **Pre-intervention period** | | **Post-intervention period** | | **Significance** |
| --- | --- | --- | --- | --- | --- |
|  | **n** | **Values** | **n** | **Value** |  |
| Door to CT (min), median (IQR) (unadjusted) | 188 | 33.0 (22.8-50.2) | 156 | 26.0 (15.0-42.0) | 0.001 |
| *Door to CT (min), median (IQR) (adjusted)* | *188* | *33.5 (29.4-37.6)* | *156* | *26.0 (22.8-29.2)* | *< 0.001* |
| Door to Needle (min), median (IQR) (unadjusted) | 149 | 102.0 (82.0-123.0) | 131 | 90.0 (67.0-113.0) | 0.005 |
| *Door to Needle (min), median (IQR) (adjusted)* | *149* | *103.7 (97.2-110.3)* | *131* | *90.3 (82.9-97.8)* | *<0.001* |
| Door-in-door-out (min), median (IQR) (unadjusted) | 36 | 148.0 (123.0-194.0) | 42 | 157.0 (119.0-189.0) | 0.55 |
| Door-in-door-out (min), median (IQR) (adjusted) | 36 | 103.9 (89.1-119) | 42 | 92.1 (73.5-111) | 0.29 |
| Door-to-arterial-access (min), median (IQR) (unadjusted) | 69 | 23.0 (9.0-53.0) | 69 | 22.0 (10.0-79.0) | 0.61 |
| Door-to-arterial-access (min), median (IQR) (adjusted) | 69 | 25.6 (8.5-42.7) | 69 | 23.2 (7.0-42.1) | 0.85 |
| 911-to-needle (min), median (IQR) (unadjusted) | 129 | 154.0 (128.0-180.0) | 94 | 151.0 (120.8-189.5) | 0.37 |
| *911-to-needle (min), median (IQR) (adjusted)* | *129* | *155.0 (145.0-165.0)* | *94* | *149.0 (137.0-161.0)* | *0.04* |
| 911-to-arterial-access (min), median (IQR) (unadjusted) | 54 | 281.0 (149.0-356.0) | 45 | 309.0 (224.0-349.0) | 0.36 |
| 911-to-arterial-access (min), median (IQR) (adjusted) | 54 | 281.0 (233.0-330.0) | 45 | 304.0 (264.0-345.0) | 0.43 |
| Length of Stay^&^ (days) median (IQR) (unadjusted) | 171 | 6 (3-11) | 145 | 6 (3-18) | 0.55 |
| Length of Stay^&^ (days) median (IQR) (adjusted) | 171 | 6.2 (5.3-7.2) | 145 | 6.3 (4.7-7.9) | 0.091 |
| *Discharged home, n (%) (unadjusted)* | *213* | *108 (50.7%)* | *179* | *47 (26.2%)* | *< 0.001* |
| *Discharged home, n (%) (adjusted)* | *213* | *109 (51.1%)* | *179* | *51 (28.5%)* | *< 0.001* |
| Discharged to rehabilitation, n (%) | 213 | 31 (14.5%) | 179 | 29 (16.2%) | 0.75 |
| Discharged to rehabilitation, n (%) (adjusted) | 213 | 31 (14.5%) | 179 | 27 (15.1%) | 0.79 |
| Mortality at discharge, n (%) (unadjusted) | 213 | 38 (17.8%) | 179 | 32 (17.8%) | 0.95 |
| Mortality at discharge, n (%) (adjusted) | 213 | 38 (17.8%) | 179 | 30 (16.7%) | 0.79 |

^&^LOS does **not include** patients that died in hospital

Table S-4: Cluster 3 - Treatment times and outcome measures for all community onset ischemic stroke patients treated with thrombolysis and/or endovascular thrombectomy. Adjustments were made for cluster, age, and sex. IQR: interquartile range, min: minutes.

|  | **Pre-intervention period** | | **Post-intervention period** | | **Significance** |
| --- | --- | --- | --- | --- | --- |
|  | **n** | **Values** | **n** | **Value** |  |
| Door to CT (min), median (IQR) (unadjusted) | 151 | 9.0 (4.0-17.0) | 83 | 12.0 (6.0-21.0) | 0.13 |
| Door to CT (min), median (IQR) (adjusted) | 151 | 8.9 (7.1-10.8) | 83 | 12.6 (9.2-16.0) | 0.063 |
| *Door to Needle (min), median (IQR) (unadjusted)* | *145* | *54.0 (40.0-75.0)* | *89* | *50.0 (31.0-73.0)* | *0.05* |
| *Door to Needle (min), median (IQR) (adjusted)* | *145* | *55.6 (49.7-61.6)* | *89* | *49.5 (41.8-57.3)* | *0.05* |
| Door-in-door-out (min), median (IQR) (unadjusted) | 0 | - | 0 | - |  |
| Door-in-door-out (min), median (IQR) (adjusted) | 0 | - | 0 | - |  |
| Door-to-arterial-access (min), median (IQR) (unadjusted) | 0 | - | 3 | 72.0 (69.5-91.0) |  |
| Door-to-arterial-access (min), median (IQR) (adjusted) | 0 | - | 3 | 72.0 (69.5-91.0) |  |
| *911-to-needle (min), median (IQR) (unadjusted)* | *99* | *103.0 (70.0-146.0)* | *40* | *87.0 (66.0-121.0)* | *0.004* |
| *911-to-needle (min), median (IQR) (adjusted)* | 99 | 100.9 (87.6-114.0) | 40 | 93.3 (80.2-106.0) | 0.04 |
| 911-to-arterial-access (min), median (IQR) | 0 | - | 0 | - |  |
| Length of Stay^&^ (days) median (IQR) (unadjusted) | 138 | 5 (3-12.50) | 84 | 7 (3-22) | 0.16 |
| Length of Stay^&^ (days) median (IQR) (adjusted) | 138 | 5 (3.8-6.1) | 84 | 7 (4.1-10) | 0.21 |
| Discharged home, n (%) (unadjusted) | 170 | 99 (58.2%) | 93 | 58 (62.3%) | 0.60 |
| Discharged home, n (%) (adjusted) | 170 | 99 (58.2%) | 93 | 59 (63.4%) | 0.42 |
| Discharged to rehabilitation, n (%) | 170 | 3 (1.76%) | 93 | 1 (1.07%) | 0.96 |
| Discharged to rehabilitation, n (%) (adjusted) | 170 | 3 (1.76%) | 93 | 1 (1.07%) | 0.96 |
| Mortality at discharge, n (%) (unadjusted) | 170 | 19 (11.17%) | 93 | 9 (9.6%) | 0.86 |
| Mortality at discharge, n (%) (adjusted) | 170 | 20 (11.7%) | 93 | 10 (10.7%) | 0.43 |

^&^LOS does **not include** patients that died in hospital

**Figure S-1: Site level improvement across each cluster**


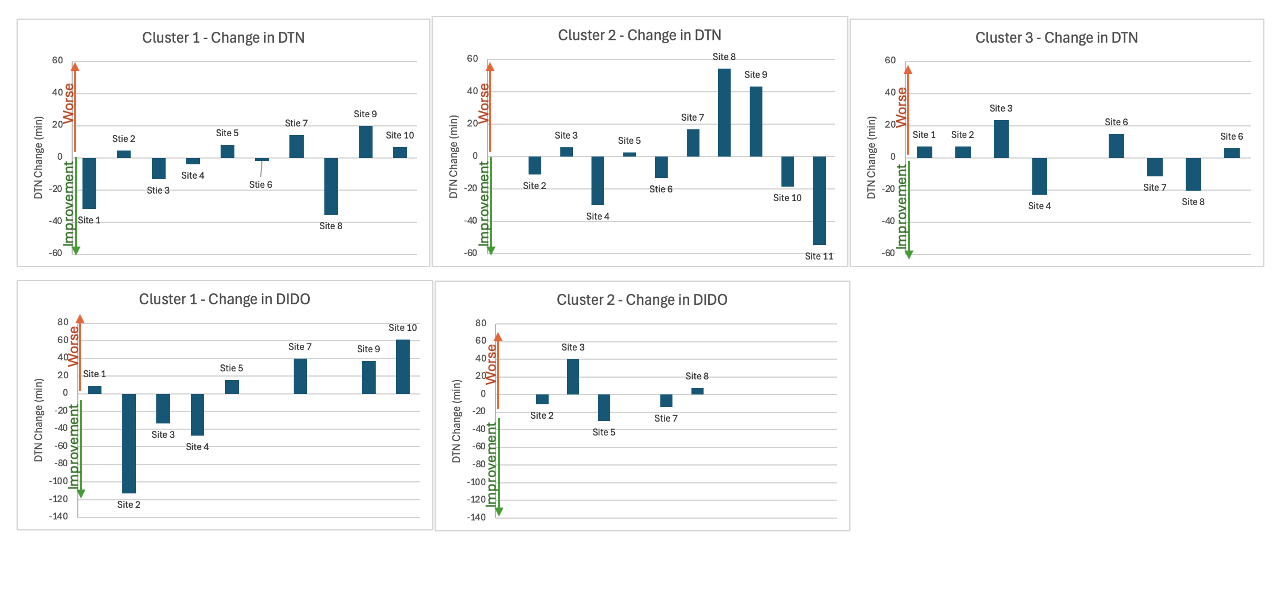


Table S-4: Sensitivity analysis of adjusting for NIHSS and not adjusting for NIHSS for treatment times and outcome measures for all community onset ischemic stroke patients treated with thrombolysis and/or endovascular thrombectomy. Adjustments were made for cluster, age, sex, and NIHSS. Adjustments* were made for cluster, age, and sex

IQR: interquartile range, min: minutes.

|  | **Control** | | **Intervention** | | **Significance** |
| --- | --- | --- | --- | --- | --- |
|  | **n** | **Values** | **n** | **Value** |  |
| Door to CT (min), median (IQR) (unadjusted) | 464 | 17.0 (9.0-36.2) | 580 | 17.0 (10.0-28.0) | 0.80 |
| Door to CT (min), median (IQR) (adjusted) | 464 | 21.9 (8.2-40.0) | 580 | 20.6 (11.6-29.3) | 0.70 |
| Door to CT (min), median (IQR) (adjusted*) | 464 | 17.3 (15.0-19.6) | 580 | 16.8 (15.1-18.4) | 0.06 |
| *Door to Needle (min), median (IQR) (unadjusted)* | *416* | *73.0 (49.0-109.0)* | *566* | *62.5 (40.0-93.0)* | *< 0.0001* |
| *Door to Needle (min), median (IQR) (adjusted)* | *416* | *71.2 (43.5-99.5)* | *566* | *62.0 (38.7-85.9)* | *0.01* |
| *Door to Needle (min), median (IQR) (adjusted*)* | *416* | *72.0 (66.1-77.9)* | *566* | *62.2(58.3-66.9)* | *0.006* |
| Door-in-door-out (min), median (IQR) (unadjusted) | 55 | 148.0 (116.0-189.5) | 91 | 155.5 (127.0-188.5) | 0.62 |
| Door-in-door-out (min), median (IQR) (adjusted) | 55 | 151.0 (100.0-202.0) | 91 | 160.0 (147.0-173.0) | 0.80 |
| Door-in-door-out (min), median (IQR) (adjusted*) | 55 | 153 (135.0-172.0) | 91 | 159.0 (148.0-169.0) | 0.59 |
| Door-to-arterial-access (min), median (IQR) (unadjusted) | 106 | 58 (11.2-101.7) | 178 | 70.0 (30.0-100.0) | 0.11 |
| Door-to-arterial-access (min), median (IQR) (adjusted) | 106 | 54.5(8.2-101.0) | 178 | 50.0 (11.0-88.0) | 0.50 |
| Door-to-arterial-access (min), median (IQR) (adjusted*) | 106 | 55.1 (41.8-68.4) | 178 | 50.4 (42.6-58.2) | 0.50 |
| 911-to-needle (min), median (IQR) (unadjusted) | 330 | 129.6 (97.0-164.6) | 413 | 125.0 (93.0-157.0) | 0.14 |
| 911-to-needle (min), median (IQR) (adjusted) | 330 | 152.0 (44.2-259.8) | 413 | 144.0 (75.4-212.6) | 0.62 |
| 911-to-needle (min), median (IQR) (adjusted*) | 330 | 131.0 (123.0-139.0) | 413 | 123.0 (116.0-130.0) | 0.15 |
| 911-to-arterial-access (min), median (IQR) (unadjusted) | 69 | 270.0 (147.0-356.0) | 88 | 273.0 (160.0-349.0) | 0.96 |
| 911-to-arterial-access (min), median (IQR) (adjusted) | 69 | 256.0 (232.0-280.0) | 88 | 239.0 (191.0-319.0) | 0.97 |
| 911-to-arterial-access (min), median (IQR) (adjusted*) | 69 | 239.0 (190.0-288.0) | 88 | 244.0 (205.0-282.0) | 0.86 |
| Length of Stay^&^ (days) median (IQR) | 423 | 6.0 (3.0-13.0) | 520 | 6.0 (3.0-14.0) | 0.30 |
| Length of Stay^&^ (days) median (IQR) (adjusted) | 423 | 5.75 (4.8-6.6) | 520 | 6.9 (4.8-9.0) | 0.16 |
| Length of Stay^&^ (days) median (IQR) (adjusted*) | 423 | 5.6 (4.9-6.2) | 520 | 6.3 (5.7-7.0) | 0.08 |
| Discharged home, n (%) | 528 | 272 (51.5%) | 673 | 282 (41.9%) | 0.001 |
| Discharged home, n (%) (adjusted) | 528 | 274 (52.0%) | 673 | 284 (42.1%) | 0.002 |
| Discharged home, n (%) (adjusted*) | 528 | 265 (50.2%) | 673 | 291 (43.2%) | 0.018 |
| Discharged to rehabilitation, n (%) | 528 | 64 (12.1%) | 673 | 107 (15.8%) | 0.07 |
| Discharged to rehabilitation, n (%) (adjusted) | 528 | 67 (12.6%) | 673 | 80 (11.8%) | 0.73 |
| Discharged to rehabilitation, n (%) (adjusted*) | 528 | 76 (14.4%) | 673 | 92 (13.7%) | 0.68 |
| Mortality at discharge, n (%) | 528 | 84 (15.9%) | 673 | 121 (17.9%) | 0.40 |
| Mortality at discharge, n(%) (adjusted) | 528 | 84 (16.0%) | 673 | 119 (17.7%) | 0.46 |
| Mortality at discharge, n(%) (adjusted) | 528 | 90 (17.0%) | 673 | 115 (17.1%) | 0.94 |
| **Cluster 1** |  |  |  |  |  |
| Door to CT (min), median (IQR) (unadjusted) | 125 | 13.0 (8.0-21.0) | 341 | 15 .0 (8.0-22.0) | 0.23 |
| Door to CT (min), median (IQR) (adjusted) | 125 | 13.0 (10.8-15.2) | 341 | 14.8 (13.5-16.0) | 0.18 |
| Door to CT (min), median (IQR) (adjusted*) | 125 | 13.0 (11.1-14.9) | 341 | 15.0 (13.7-16.3) | 0.32 |
| Door to Needle (min), median (IQR) (unadjusted) | 122 | 57.0 (41.5-83.5) | 346 | 58.0 (37.0-83.0) | 0.79 |
| Door to Needle (min), median (IQR) (adjusted) | 122 | 57.2 (50.7-63.8) | 346 | 58.5 (54.5-62.5) | 0.70 |
| Door to Needle (min), median (IQR) (adjusted*) | 122 | 58.0 (51.7-64.2) | 346 | 58.4 (54.4-62.4) | 0.89 |
| Door-in-door-out (min), median (IQR) (unadjusted) | 19 | 137.0 (114.18-212) | 49 | 153.0 (134.0-187.0) | 0.62 |
| Door-in-door-out (min), median (IQR) (adjusted) | 19 | 136.8 (103.6-169.9) | 49 | 155.0 (134.6-175.6) | 0.36 |
| Door-in-door-out (min), median (IQR) (adjusted*) | 19 | 163.0 (122-203.0) | 49 | 152 (133.0-172.0) | 0.44 |
| Door-to-arterial-access (min), median (IQR) (unadjusted) | 37 | 86.0 (56.0-123.0) | 106 | 77.5 (63.0-101.8) | 0.42 |
| Door-to-arterial-access (min), median (IQR) (adjusted) | 37 | 85.7 (69.6-101.7) | 106 | 77.0 (67.7-86.5) | 0.37 |
| Door-to-arterial-access (min), median (IQR) (adjusted*) | 37 | 85.1 (62.4-107.9) | 106 | 75.7 (68.0-83.5) | 0.21 |
| 911-to-needle (min), median (IQR) (unadjusted) | 102 | 113.9 (89.5-145.3) | 279 | 119.2 (93.8-153.3) | 0.30 |
| 911-to-needle, median (IQR) (adjusted) | 102 | 113.4 (101.0-125.6) | 279 | 118.5 (111.0-125.9) | 0.40 |
| 911-to-needle, median (IQR) (adjusted*) | 102 | 112.0 (98.1-126.0) | 279 | 118.0 (110.8-125) | 0.42 |
| 911-to-arterial-access (min), median (IQR) (unadjusted) | 15 | 213.0 (146.1-332.1) | 43 | 180.0 (135.9-349.1) | 0.67 |
| 911-to-arterial-access (min), median (IQR) (adjusted) | 15 | 236.0 (131.0-341.0) | 43 | 203.0 (165.0-241.0) | 0.42 |
| 911-to-arterial-access (min), median (IQR) (adjusted*) | 15 | 182.0 (80.4-283.0) | 43 | 180.0 (94.0-266.0) | 0.97 |
| Length of Stay (days), median (IQR)^&^ (unadjusted) | 114 | 6 (3-14) | 300 | 6 (3-13) | 0.67 |
| Length of Stay (days), median (IQR)^&^ (adjusted) | 114 | 6.7 (4.7-8.6) | 300 | 6.6 (5.5-7.8) | 0.90 |
| Length of Stay (days), median (IQR)^&^ (adjusted*) | 114 | 5.2 (3.7-6.6) | 300 | 6.3 (5.4-7.1) | 0.16 |
| Discharged home, n (%) (unadjusted) | 145 | 65 (44.8%) | 401 | 177 (44.1%) | 0.80 |
| Discharged home, n (%) (adjusted) | 145 | 63 (43.7%) | 401 | 179 (44.7%) | 0.35 |
| Discharged home, n (%) (adjusted*) | 145 | 66 (45.5%) | 401 | 175 (43.6%) | 0.66 |
| Discharged to rehabilitation, n (%) | 145 | 30 (20.6%) | 401 | 77 (19.2%) | 0.79 |
| Discharged to rehabilitation, n (%) (adjusted) | 145 | 31 (21.6%) | 401 | 75 (18.7%) | 0.44 |
| Discharged to rehabilitation, n (%) (adjusted*) | 145 | 31 (21.3%) | 401 | 76 (18.9%) | 0.60 |
| Mortality at discharge, n (%) (unadjusted) | 145 | 27 (18.6%) | 401 | 81 (20.1%) | 0.68 |
| Mortality at discharge, n (%) (adjusted) | 145 | 27 (18.9%) | 401 | 80 (20.4%) | 0.70 |
| Mortality at discharge, n (%) (adjusted) | 145 | 26 (17.9%) | 401 | 82 (20.4%) | 0.50 |
| **Cluster 2** |  |  |  |  |  |
| Door to CT (min), median (IQR) (unadjusted) | 188 | 33.0 (22.8-50.2) | 156 | 26.0 (15.0-42.0) | 0.001 |
| *Door to CT (min), median (IQR) (adjusted)* | *188* | *32.5 (28.6-36.2)* | *156* | *25.0 (22.4-29.6)* | *0.009* |
| *Door to CT (min), median (IQR) (adjusted*)* | *188* | *33.5 (29.4-37.6)* | *156* | *26.0 (22.8-29.2)* | *< 0.0001* |
| Door to Needle (min), median (IQR) (unadjusted) | 149 | 102.0 (82.0-123.0) | 131 | 90.0 (67.0-113.0) | 0.005 |
| *Door to Needle (min), median (IQR) (adjusted)* | *149* | *102.2 (95.0-109.0)* | *131* | *89.6 (83.0-97.0)* | *0.01* |
| *Door to Needle (min), median (IQR) (adjusted*)* | *149* | *103.7 (97.2-110.3)* | *131* | *90.3 (82.9-97.8)* | *<0.001* |
| Door-in-door-out (min), median (IQR) (unadjusted) | 36 | 148.0 (123.0-194.0) | 42 | 157.0 (119.0-189.0) | 0.55 |
| Door-in-door-out (min), median (IQR) (adjusted) | 36 | 146.8 (127.0-167.0) | 42 | 162.6 (144.0-181.0) | 0.26 |
| Door-in-door-out (min), median (IQR) (adjusted*) | 36 | 103.9 (89.1-119) | 42 | 92.1 (73.5-111) | 0.29 |
| Door-to-arterial-access (min), median (IQR) (unadjusted) | 69 | 23.0 (9.0-53.0) | 69 | 22.0 (10.0-79.0) | 0.61 |
| Door-to-arterial-access (min), median (IQR) (adjusted) | 69 | 27.2 (10.0-44.0) | 69 | 21.6 (4.0-38.0) | 0.65 |
| Door-to-arterial-access (min), median (IQR) (adjusted*) | 69 | 25.6 (8.5-42.7) | 69 | 23.2 (7.0-42.1) | 0.85 |
| 911-to-needle (min), median (IQR) (unadjusted) | 129 | 154.0 (128.0-180.0) | 94 | 151.0 (120.8-189.5) | 0.37 |
| 911-to-needle (min), median (IQR) (adjusted) | 129 | 151.6 (143.4-159.7) | 94 | 146.0 (136.5-155.6) | 0.38 |
| 911-to-needle (min), median (IQR) (adjusted*) | 129 | 155 (145.0-165.0) | 94 | 149.0 (137.0-161.0) | 0.04 |
| 911-to-arterial-access (min), median (IQR) (unadjusted) | 54 | 281.0 (149.0-356.0) | 45 | 309.0 (224.0-349.0) | 0.36 |
| 911-to-arterial-access (min), median (IQR) (adjusted) | 54 | 280.4 (240.7-320.1) | 45 | 307.1 (263.6-350.6) | 0.37 |
| 911-to-arterial-access (min), median (IQR) (adjusted*) | 54 | 281.0 (233.0-330.0) | 45 | 304.0 (264.0-345.0) | 0.43 |
| Length of Stay^&^ (days) median (IQR) (unadjusted) | 171 | 6 (3-11) | 145 | 6 (3-18) | 0.55 |
| Length of Stay^&^ (days) median (IQR) (adjusted) | 171 | 6.9 (5.5-8.3) | 145 | 7.6 (5.7-9.4) | 0.60 |
| Length of Stay^&^ (days) median (IQR) (adjusted*) | 171 | 6.2 (5.3-7.2) | 145 | 6.3 (4.7-7.9) | 0.091 |
| *Discharged home, n (%) (unadjusted)* | *213* | *108 (50.7%)* | *179* | *47 (26.2%)* | *< 0.0001* |
| *Discharged home, n (%) (adjusted)* | *213* | *110 (51.6%)* | *179* | *49 (27.3%)* | *0.01* |
| *Discharged home, n (%) (adjusted*)* | *213* | *109 (51.1%)* | *179* | *51 (28.5%)* | *< 0.0001* |
| Discharged to rehabilitation, n (%) | 213 | 31 (14.5%) | 179 | 29 (16.2%) | 0.75 |
| Discharged to rehabilitation, n (%) (adjusted) | 213 | 31 (14.8%) | 179 | 28 (15.6%) | 0.91 |
| Discharged to rehabilitation, n (%) (adjusted*) | 213 | 31 (14.5%) | 179 | 27 (15.1%) | 0.79 |
| Mortality at discharge, n (%) (unadjusted) | 213 | 38 (17.8%) | 179 | 32 (17.8%) | 0.95 |
| Mortality at discharge, n (%) (adjusted) | 213 | 36 (16.9%) | 179 | 32 (17.8%) | 0.90 |
| Mortality at discharge, n (%) (adjusted) | 213 | 38 (17.8%) | 179 | 30 (16.7%) | 0.79 |
| **Cluster 3** |  |  |  |  |  |
| Door to CT (min), median (IQR) (unadjusted) | 151 | 9.0 (4.0-17.0) | 83 | 12.0 (6.0-21.0) | 0.13 |
| Door to CT (min), median (IQR) (adjusted) | 151 | 9.6 (8.0-12.0) | 78 | 11.8 (9.0-15.0) | 0.24 |
| Door to CT (min), median (IQR) (adjusted*) | 151 | 8.9 (7.1-10.8) | 83 | 12.6 (9.2-16.0) | 0.063 |
| *Door to Needle (min), median (IQR) (unadjusted)* | *145* | *54.0 (40.0-75.0)* | *89* | *50.0 (31.0-73.0)* | *0.05* |
| *Door to Needle (min), median (IQR) (adjusted)* | *145* | *54.5 (44.0-67.0)* | *89* | *49.0 (41.7-56.3)* | *0.05* |
| *Door to Needle (min), median (IQR) (adjusted*)* | *145* | *55.6 (49.7-61.6)* | *89* | *49.5 (41.8-57.3)* | *0.05* |
| Door-in-door-out (min), median (IQR) (unadjusted) | 0 | - | 0 | - |  |
| Door-to-arterial-access (min), median (IQR) (unadjusted) | 0 | - | 3 | 72.0 (69.5-91.0) |  |
| Door-to-arterial-access (min), median (IQR) (adjusted) | 0 | - | 3 | 72.0 (69.5-91.0) |  |
| Door-to-arterial-access (min), median (IQR) (adjusted*) | 0 | - | 3 | 72.0 (69.5-91.0) |  |
| *911-to-needle (min), median (IQR) (unadjusted)* | *99* | *103.0 (70.0-146.0)* | *40* | *87.0 (66.0-121.0)* | *0.004* |
| *911-to-needle (min), median (IQR) (adjusted)* | *99* | *102.8 (90.9-114.7)* | *40* | *90.1 (70.9-109.3)* | *0.05* |
| 911-to-needle (min), median (IQR) (adjusted*) | 99 | 100.9 (87.6-114.0) | 40 | 93.3 (80.2-106.0) | 0.04 |
| 911-to-arterial-access (min), median (IQR) | 0 | - | 0 | - |  |
| Length of Stay^&^ (days) median (IQR) (unadjusted) | 138 | 5 (3-12.50) | 84 | 7 (3-22) | 0.16 |
| Length of Stay^&^ (days) median (IQR) (unadjusted) | 138 | 5 (3-12.50) | 84 | 7 (3-22) | 0.16 |
| Length of Stay^&^ (days) median (IQR) (adjusted*) | 138 | 5 (3.8-6.1) | 84 | 7 (4.1-10) | 0.21 |
| Discharged home, n (%) (unadjusted) | 170 | 99 (58.2%) | 93 | 58 (62.3%) | 0.60 |
| Discharged home, n (%) (adjusted) | 170 | 100 (59.0%) | 93 | 57 (61.2%) | 0.79 |
| Discharged home, n (%) (adjusted*) | 170 | 99 (58.2%) | 93 | 59 (63.4%) | 0.42 |
| Discharged to rehabilitation, n (%) | 170 | 3 (1.76%) | 93 | 1 (1.07%) | 0.96 |
| Discharged to rehabilitation, n (%) (adjusted) | 170 | 3 (1.76%) | 93 | 1 (1.07%) | 0.96 |
| Discharged to rehabilitation, n (%) (adjusted*) | 170 | 3 (1.76%) | 93 | 1 (1.07%) | 0.96 |
| Mortality at discharge, n (%) (unadjusted) | 170 | 19 (11.17%) | 93 | 9 (9.6%) | 0.86 |
| Mortality at discharge, n (%) (adjusted) | 170 | 17 (10.2%) | 93 | 11 (11.8%) | 0.80 |
| Mortality at discharge, n (%) (adjusted) | 170 | 20 (11.7%) | 93 | 10 (10.7%) | 0.43 |
